# Use of hydrocolloid dressing in infants requiring open chest management after cardiac surgery

**DOI:** 10.1186/s40792-021-01330-4

**Published:** 2021-11-22

**Authors:** Shinya Yokoyama, Rei Tonomura, Ryohei Fukuba, Kazuhiro Mitani, Hideki Uemura

**Affiliations:** Congenital Heart Disease Center, Division of Cardio-Thoracic Surgery, Nara Medical University, 840, Shijo-cho, Kashihara, Nara 634-8522 Japan

**Keywords:** Congenital heart surgery, Neonate, Infant, Delayed sternal closure, Hydrocolloidal wound dressing, Wound healing

## Abstract

**Purpose:**

Sternal splintage is known as an effective maneuver to stabilize hemodynamics during the immediate postoperative period, particularly in very sick infants. On the other hand, its wound management is not always straightforward. We employed dressing using a product made of a hydrocolloid material in such circumstances. This report describes our experience in utilizing the dressing in term of its potential advantages.

**Materials and methods:**

Six infants needed open chest management following complicated procedures for congenital heart disease. A polytetrafluoroethylene patch was fixed to augment the skin defect at the time of sternal splintage, and a hydrocolloid dressing was applied to entirely cover the wound including the suture line.

**Result:**

All patients survived their difficult circumstances. None of them suffered wound complications such as infection or healing problem during sternal splintage or subsequent to eventual chest closure. The dressing product was easy to handle with no adverse events associated with its material.

**Conclusions:**

It is reconfirmed that a dressing made of hydrocolloid material was of practical use for sealing the wound in infants requiring open chest management after cardiac surgery.

## Introduction

The sternum might not be closed primarily in very sick neonates and infants following cardiac procedures for congenital heart disease in particular when extracorporeal circulation was needed. In those occasions, the sternum is splinted, and the wound is temporarily covered with an augmentation patch until the chest is to be closed several days later. Usually, a water-repellent film is used, as a dressing, to seal the patch and its suture line against bacterial infection through the area. We report our experience of adopting a hydrocolloid dressing for this purpose.

## Materials and methods

**Fig. 1:**
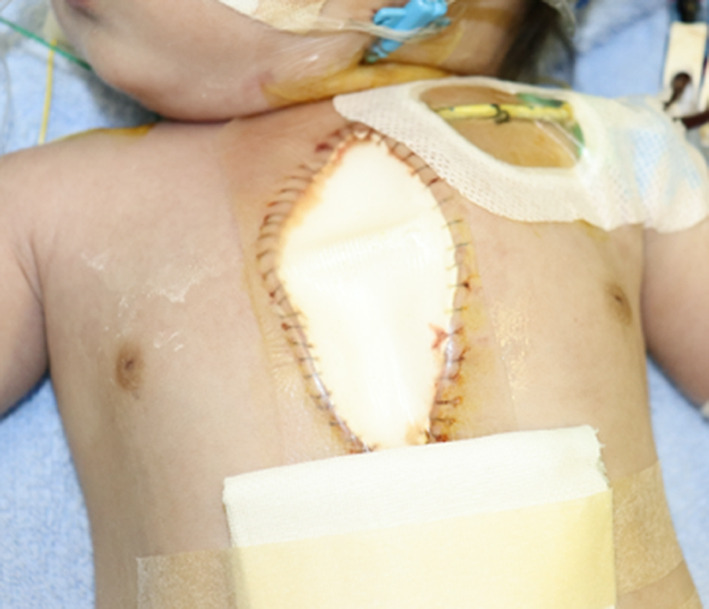
A hydrocolloid wound dressing was applied to entirely cover the wound and the suture line

We encountered five neonates and a 2-month-old infant who required sternal splintage following open-heart surgery between December 2018 and December 2020 (Table 1). In these 6 patients, a hydrocolloid dressing (Karayahesive®; Alcare, Tokyo, Japan) was chosen to entirely cover the wound (Fig. 1). No rods were applied to splint the sternum. Prior to dressing, the skin had been augmented using a 1 mm-thick PTFE patch; the patch being entirely fixed to the dermis with 5–0 polypropylene sutures in a contentious way. The subcutaneous layer was not open to the air at all. In a patient in whom a central ECMO was applied, the arterial and the venous cannulas were situated to the cranial and the caudal margins of the wound, respectively, and independently surrounded by the dermis there. Thus, the wound was made entirely air-tight, including the sites around the cannulas, and placement of a “Karayahesive” dressing reinforced the seal.

**Table 1:** Patients’ characteristics

Patient	Gender	Age (days)	Diagnosis	Surgical procedures	ECC time (min)	AoX time (min)	Duration of SS (days)	Dressing exchange (times)
3	F	0	TAPVC (1b), severe PVO	TAPVC repair	257	91	4	0
1	F	16	TGA, CoA, PDA	Arterial switch + CoA repair	507	305	3	0
4	F	16	Right isomerism, single RV, DORV, pulmonary atresia, nonconfluent PA, CAVV regurgitation (moderate), bilateral PDA,TAPVC (2b)	CAVV repair + PA reconstruction + central shunt (φ3.5 mm) + ECMO	505	206	5	2
5	F	17	DORV (non-committed VSD), ASD, PDA, right aortic arch	Arterial switch + Intraventricular rerouting + ASD closure	469	312	3	0
2	M	18	HLHS (mitral & aortic atresia)	Norwood	329	98	4	0
6	F	84	Single LV, restrictive ASD, TR(severe), MR(moderate), SAS, after PA banding	ASD creation + tricuspid valve closure + PA angioplasty + DKS anastomosis + BDG	349	206	7	1

ECC, extracorporeal circulation; AoX, aortic cross clamp; SS, sternal splintage; TGA, transposition of the great arteries; CoA, coarctation of the aortic arch; PDA, patent ductus arteriosus; HLHS, hypoplastic left heart syndrome; TAPVC, totally anomalous pulmonary venous connection; PVO, pulmonary venous obstruction; RV, right ventricle; DORV, double outlet right ventricle; PA, pulmonary artery; CAVV, common atrio-ventricular valve; ASD, atrial septal defect; LV, left ventricle; TR, tricuspid valve regurgitation; MR, mitral valve regurgitation; SAS, subaortic stenosis; ASO, arterial switch operation; ECMO, extracorporeal membrane oxygenation; DKS, Damus-Kaye-Stansel; BDG, bidirectional Glenn procedure

Open chest management was considered necessary because of (1) systolic arterial pressure staying below 50 mmHg, (2) central venous pressure going above 15 mmHg, (3) frequent arrhythmia, (4) intractable drop of blood oxygen saturation, subsequent to attempted primary sternal closure, or (5) staying on ECMO.

In cases 4 and 6, the hydrocolloid dressing was renewed at the time of coming off ECMO and removal of a direct RA line, respectively. In the other 4 patients, the initial dressing was kept untouched. Daily sterilizing maneuver was skipped to the region under the hydrocolloid material.

## Results

Patients’ characteristics are shown in Table 1. All survived their difficult postoperative period. They are eventually doing well. No complications occurred, such as wound infection, mediastinitis, contact dermatitis, or skin necrosis. No scar revision has been needed in the longer terms. Air leakage was seen in none of the patients through the drainage tubes. We judged that the hydrocolloid dressing was sufficiently air-tight. Leachate did not come out of the affixed area until eventual chest closure 3–7 (4.3 ± 1.5) days after the initial procedure.

## Discussion

It is well known that sternal splintage is effective to maintain stable postoperative circulation particularly in neonates and infants with complicated cardiac condition. The heart wound not function appropriately if squeezed by the swollen tissue around. Edema of those tissues as well as that of the myocardium itself does improve usually within several days after surgery. In this respect, delayed chest closure is sensible. Still, this period of days, although short, can be troublesome in terms of wound healing and undesired infection. The surgeons and the nurses are strived to optimize wound care for better outcome.

Film dressings are commonly used to cover the anterior chest. They may not be sticky enough to perfectly separate the wound from the air. In some instances, the dressing could come off the skin, or leachate inside could flow out through a tiny fold of the patch or the dressing. On the other hand, the skin may be irritated with dermatitis caused by adhesive agents.

**Fig. 2:**
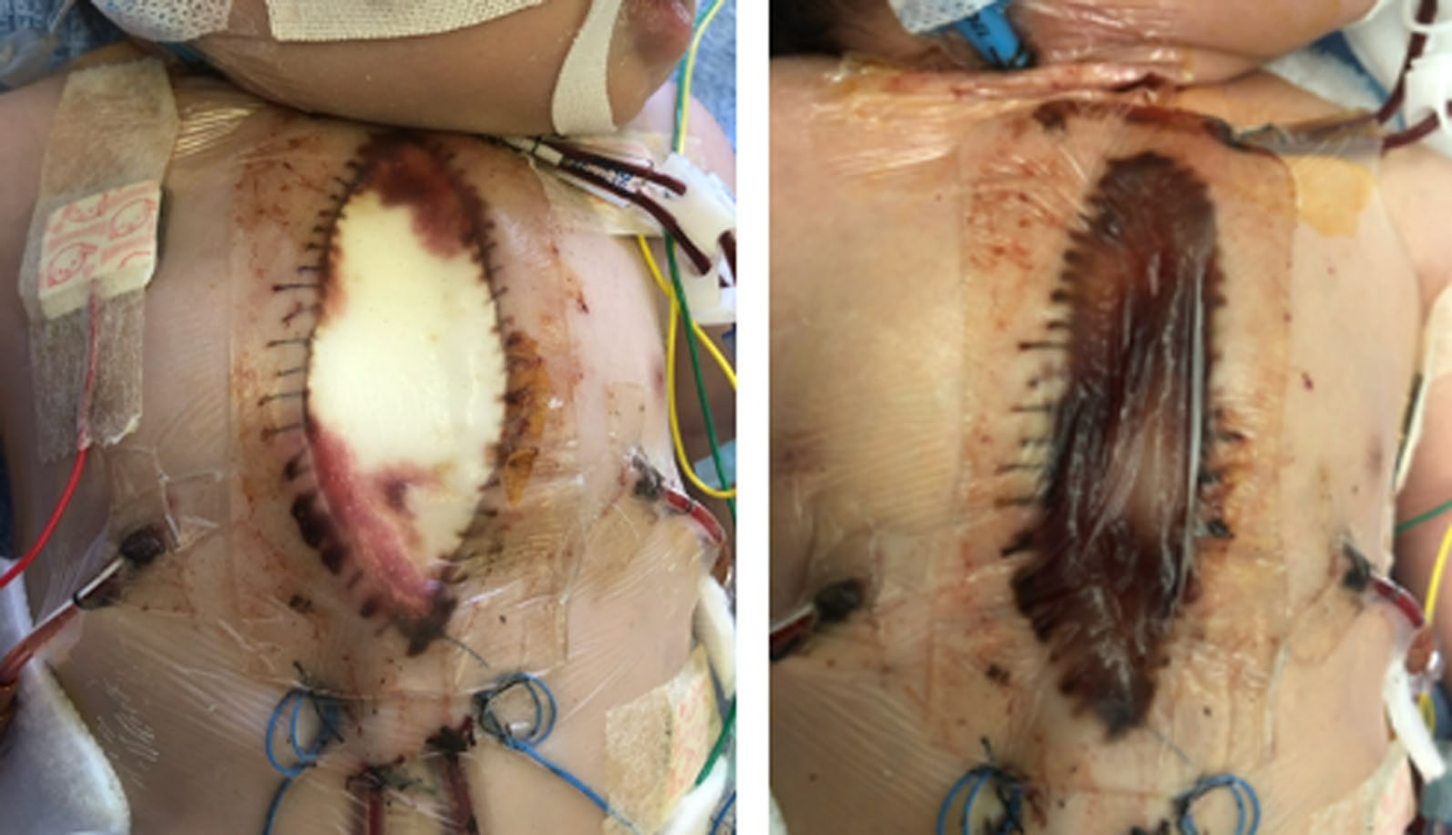
The hydrophilic material initially white (left: one day after surgery) absorbed leachate liquid and turned into gel (right: 3 days after surgery)

Karayahesive® is, allegedly, a material coated with hydrocolloid, consisting of two layers: a skin-adhesive layer made from Karaya gum to maintain a moist environment, and an outer layer of polyurethane film to provide a waterproof barrier against the outside the body. On the basis of our experience, this product is flexible and easy to handle. Also, sufficiently self-adhesive. Liquid is absorbed in its hydrophilic layer and turning into gel, which is advantageous for wound management avoiding overflow of leachate from the wound (Fig. 2). We can readily check the condition under the dressing since the material is see-through. Basically, the dressing does not need renewal for several days. This is advantageous in terms of cost-performance [1, 2]. In addition, Karayahesive No. 6 costs 40% of the smallest product of an Isodine drape (285 versus 719 Japanese Yen per sheet). An Isodine drape inadequately adhesive comes off sometimes and needs to be reupholstered; this makes the cost even higher.

It was an easy matter to remove the Karayahesive® used; just peeled off. Because moisture had been maintained at the skin edges, the tissues there remained in a good condition without necrosis or dried-up damage. Debridement of the wound margin was not necessary.

Some articles have been published reporting use of this material in cardiac surgery [3–5]. We focused on its use in delayed sternal closure. Our current experience enhances the opinions previously described. Being nicely air-tight, appropriately adhesive, easy to handle, and moisture controlling, this material appeared to assist wound healing and protect against bacterial infection. We would not hesitate to employ this material in a similar circumstance.

## Conclusions

We reconfirm that a dressing made of hydrocolloid material was of practical use for sealing the wound in infants requiring open chest management after cardiac surgery.

## Data Availability

Not applicable.

## Abbreviations

PTFE: Polytetrafluoroethylene
ECMO: Extracorporeal membranous oxygenation
RA: Right atrium
